# Acute neurological care in north-east Germany with telemedicine support (ANNOTeM): protocol of a multi-center, controlled, open-label, two-arm intervention study

**DOI:** 10.1186/s12913-020-05576-w

**Published:** 2020-08-17

**Authors:** J. E. Weber, A. Angermaier, K. Bollweg, H. Erdur, S. Ernst, A. Flöel, C. Gorski, F. I. Kandil, S. Kinze, K. Kleinsteuber, T. Kurth, I. Schmehl, S. Theen, M. Endres, H. J. Audebert, for the ANNOTeM-consortium

**Affiliations:** 1 Klinik und Hochschulambulanz für Neurologie, Charité – Universitätsmedizin Berlin, Hindenburgdamm 30, D-12203 Berlin, Germany; 2 Center for Stroke Research Berlin (CSB), Charité – Universitätsmedizin Berlin, Berlin, Germany; 3 Clinical Research Unit, Berlin Institute of Health, Berlin, Germany; 4 Department of Neurology, University Medicine Greifswald, Greifswald, Germany; 5 German Center for Neurodegenerative Diseases, partner site, Rostock, Greifswald Germany; 6 Institute of Public Health, Charité – Universitätsmedizin Berlin, Berlin, Germany; 7 Institute for Biometry and Clinical Epidemiology, Charité – Universitätsmedizin Berlin, Berlin, Germany; 8 Unfallkrankenhaus Berlin, Berlin, Germany; 9 Excellence Cluster NeuroCure, Berlin, Germany; 10 German Center for Neurodegenerative Diseases (DZNE), partner site Berlin, Berlin, Germany; 11 German Centre for Cardiovascular Research (DZHK), partner site Berlin, Berlin, Germany

**Keywords:** ANNOTeM, Emergency medicine, Health care research, Neuro acute units, Quality management, Stroke / Neurological disease, Telemedicine

## Abstract

**Background:**

Both diagnosis and treatment of neurological emergencies require neurological expertise and are time-sensitive. The lack of fast neurological expertise in regions with underserved infrastructure poses a major barrier for state-of-the-art care of patients with acute neurological diseases and leads to disparity in provision of health care. The main purpose of ANNOTeM (acute neurological care in North East Germany with telemedicine support) is to establish effective and sustainable support structures for evidence based treatments for stroke and other neurological emergencies and to improve outcome for acute neurological diseases in these rural regions.

**Methods:**

A “hub-and-spoke” network structure was implemented connecting three academic neurological centres (“hubs”) and rural hospitals (“spokes”) caring for neurological emergencies. The network structure includes (1) the establishment of a 24/7 telemedicine consultation service, (2) the implementation of standardized operating procedures (SOPs) in the network hospitals, (3) a multiprofessional training scheme, and (4) a quality management program. Data from three major health insurance companies as well as data from the quality management program are being collected and evaluated. Primary outcome is the composite of first time of receiving paid outpatient nursing care, first time of receiving care in a nursing home, or death within 90 days after hospital admission.

**Discussion:**

Beyond stroke only few studies have assessed the effects of telemedically supported networks on diagnosis and outcome of neurological emergencies. ANNOTeM will provide information whether this approach leads to improved outcome. In addition, a health economic analysis will be performed.

**Study registration:**

German Clinical Trials Register DRKS00013067, date of registration: November 16 th, 2017, URL: http://www.drks.de/DRKS00013068

## Background

The implementation of stroke units [1] along with the advent of intravenous thrombolysis [1] as well as thrombectomy of large vessel occlusion [2] has led to a huge leap in evidence-based acute stroke therapy. However, treatment effects are time-sensitive and depend on swift application of specific therapies. This is not only true for stroke but also for other neurological emergencies like seizures, meningoencephalitis, and spinal cord injury, where fast and effective diagnostication and treatment have been shown to improve outcome [3–5]. Neurological emergencies are a major cause of functional dependence [6]. Hospitals with emergency departments but without a neurology sercive may struggle to provide up-to-date care for patients with neurological emergencies. This applies particularly to rural and other regions with underserved infrastructure. Unfortunately, most neurological diseases peak at higher age, which means that rural areas with typically elderly populations face a particular challenge in providing appropriate care.

Telemedicine networks offer the potential for providing neurological expertise for emergency departments without expert neurologists [7]. In fact, neurological diseases can be adequately assessed via audiovisual examination because typical neurological deficits like palsies, disturbances of speech or consciousness can be reliably captured via video stream [8, 9] and brain imaging is easily transferable digitally.

Telestroke networks have been developed as a blueprint for teleneurological treatment during recent years. Treatment of acute stroke using telemedicine resulted in more frequent and appropriate application of intravenous thrombolysis [10, 11] and was associated with improved outcome [12]. What is more, “telestroke” proved to be cost-effective [13] making stroke one of the most frequent indications for telemedicine in several countries (Refs als from US, Germany etc).

### Aims

ANNOTeM (acute neurological care in North-East Germany with telemedicine support) aims to improve health care by use of telemedicine for acute time-dependent neurological diseases beyond stroke, in particular coma/status epilepticus, meningoencephalitis, and spinal cord injury, and therefore establish sustainable structures for implementation of evidence based treatments in underserved areas in North-East Germany.

## Methods/design

### Hypothesis

A multi-component intervention with telemedicine support for neurological emergency care extends time to death or first time of receiving paid outpatient nursing care or first time of receiving care in a nursing home within 90 days after hospital admission.

### Study design and setting

ANNOTeM is a multi-center, controlled, open-label, two-arm intervention study. Primary and secondary outcomes will be evaluated for pre- and post-implemention of the network. The ANNOTEM intervention started in November 2017 and is expected to run until the end of July 2020.

### Conventional care

Community hospitals, except one without a neurology department, were included in the teleneurological network if they had treated a significant number of at least 50 patients per year with main discharge diagnosis of an acute neurological disease. During the control period, patients had usually been admitted to the internal medicine department and had been seen by a neurologist the following day at the earliest. Typically, management of acute neurological diseases had not been based on standardized operating procedures (SOP) in these hospitals prior to the project.

### Intervention

#### Structure of the network

The teleneurological network ANNOTeM consists of a consortium of four neurocenters (Charité -Universitätsmedizin Berlin, Universitätsmedizin Greifswald, Unfallkrankenhaus Berlin, Epilepsieklinik Tabor) with neurological and epileptological expertise, three health insurance companies (Allgemeine Ortskrankenkasse Nordost, Potsdam (AOK Nordost), Barmer Ersatzkasse, Wuppertal (BARMER), Techniker Krankenkasse, Hamburg (TK)), one technology partner specialized on telemedicine communication products (MEYTEC, Werneuchen, Germany) and the Institute of Public Health, Charité -Universitätsmedizin Berlin,as evaluating institute. The consortium is led by the Charité - Universitätsmedizin Berlin. The clinical centers provide teleneurological care to 11 regional hospitals in the northeastern federal states of Germany (2 in Mecklenburg-Western Pomerania, 8 in Brandenburg and one in Saxony-Anhalt). One regional hospital runs its own neurological department with a certified regional Stroke Unit. In the ten hospitals, the “Neuro Acute Units” were set up as part of the intervention and managed by the respective departments of internal medicine in nine hospitals, whereas in the remaining hospital the “Neuro Acute Unit” is managed as part of an interdisciplinary intensive care unit. In addition, the network cooperates with other neurological, neuroradiological and neurosurgical facilities in the region.

#### Neuro-acute-units

The structure of the “Neuro-Acute-Units” is based on the concept of the German Tele-Stroke-Units [14] and aims at specialized treatment for a broader spectrum of acute neurological diseases. In addition to the standards already defined for Tele-Stroke Units, quality management, training, courses, and bed-side visits will be extended to the management other acute neurological diseases considered as part of the intervention for physicians, nurses and therapists.

#### Implementation of standardized operating procedures

Standardized operating procedures (SOP) for management of acute stroke, epileptic seizures/epilepsy, meningitis/encephalitis, and acute spinal cord injury were developed by consensus of the four neurocenters [15, 16]. These SOPs are generally based on the guidelines of the German Neurological Society (Deutsche Gesellschaft für Neurologie, DGN) und Joint Workgroup of German Medical Societies (Arbeitsgemeinschaft der Wissenschaftlichen Medizinischen Fachgesellschaften, AWMF), but had to be adapted in some aspects with regard to the telemedicine setting. Furthermore, they undergo regular updates according to the evolving scientific evidence. Before initiating teleconsultations, each SOP was presented to each team of the network hospitals followed by a discussion of their implementation in clinical routine.

#### Quality management

With start of the network, each cooperating community hospital started documentation of quality indicators within the quality assessment for stroke in Northwest Germany (*Qualitätssicherung Schlaganfall Nordwestdeutschland*, based on the *Arbeitsgemeinschaft Deutschsprachiger Schlaganfall Register* (ADSR)). The ANNOTeM network developed additional quality assessment instruments for other acute neurological diseases (epilepsy, meningitis/encephalitis, and spinal cord injury) for their use in the regional hospitals.

In addition to quality assessment, regular meetings on treatment quality are held in the cooperating regional hospitals with mutual discussion of the proportions of patients presented via telemedicine, documentation rates as well as the SOP-adherence and interhospital transfers of patients.

#### Teleneurological consultations

**Fig. 1:**
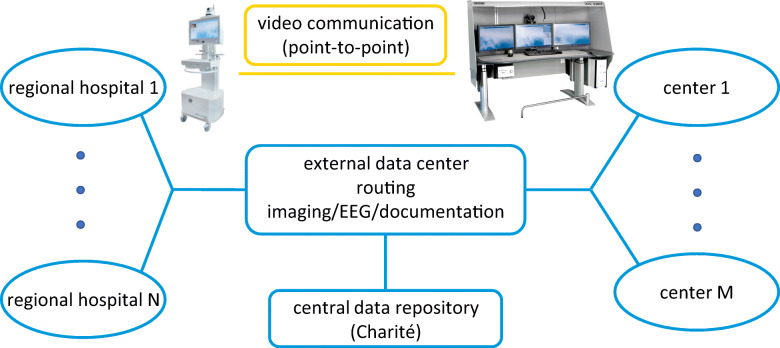
Representation of data flows in the telemedical network ANNOTeM. The video data is transmitted as a stream using a point-to-point connection, while the documentation and other examination data are stored in a central repository. With permission of MEYTEC GmbH, the telemedical equipment used in ANNOTeM is shown (left: VIMED TELEDOC, right: VIMED DOC)

Teleneurological consultations are provided by physicians who are experienced in stroke treatment and meet the standard of board certified neurologists for diagnosis and treatment of neurological emergencies. The consultation service is staffed 24/7 by a full-time physician. All physicians working in the telemedicine service receive a practice training for the technical system as well as for the procedures of teleneurological examination and treatment of patients. The cooperating regional hospitals can present all patients with acute neurological symptoms in teleconsultation. For this purpose, a hotline is used to contact the telemedicine centers. The patient is examined via video conference with remote-controlled camera together with the onsite physician. In addition, there is a connection to the imaging data systems of the cooperating regional hospitals. This can be used to forward any computer tomography (CT) or magnetic resonance imaging (MRI) images to a central Picture Archiving and Communication System (PACS), accessible from the teleneurology hubs. The data transfer in ANNOTeM is realized as a point-to-point connection between the respective active neurocentres and the regional cooperation hospitals (see Fig. 1). All connections are encrypted according to state-of-the-art data protection requirements (GDPR, General Data Protection Rules). Data are stored on secured servers with access restricten to staff of the medical project management. Video streams are not stored. After each consultation, a written statement with recommendations for acute treatment and further diagnostic procedures is sent electronically (PDF) to the regional hospital and printed automatically. During the monitoring on the neuro-acute unit, a board certified neurologist sees the patient during ward rounds on a daily basis.

#### Transfer management

Patients who need specialized treatment beyond the capacities of the implemented “Neuro-Acute-Units” (in particular patients with need for neuroradiological or neurosurgical treatment, not being available in regional network hospitals) are transferred to the nearest suitable hospital. In order to avoid delays in the referral hospital, point-to-point connections have been established between the telemedicine centres and the various referral hospitals, allowing the transmission of imaging data collected in network hospitals. In addition, referral hospitals report data on processes and in-hospital outcomes for the respective patients to the coordinating center.

### Study population

#### Inclusion criteria

To determine the composite primary outcome of time to death or first-time care at home or paid care support for the first time within 90 days after the index event, we will use routine data from three cooperating health insurance companies (AOK Nordost, BARMER, TK). For this purpose, eligible study participants must (1) have continuous health insurance with one of these health insurers during the evaluation period, (2) be admitted during the intervention or control period to one of the cooperating regional hospitals, (3) have a neurological diagnosis (International Classification of Diseases (ICD)-10 main diagnosis) such as stroke (I60–64), status epilepticus or impaired level of consciousness (G41, R40), meningitis /encephalitis (A82.0–2, A85–87, G00–03, G06–09), acute spinal cord injury (S14, S24, S34, G82), (4) and be over 18 years of age of both sexes.

We had originally planned to include patients diagnosed with traumatic brain injury (S06–07) in an earliert version of the study protocoll. However, since patients with traumatic brain injury are only rarely managed by our project partners in the participating regional hospitals they are not included in the primary study population, but rather as a companion study population in a sensitivity analysis.

#### Intervention group (post-implementation)

The intervention group consists of patients treated in one of the eleven cooperating regional hospitals diagnosed with any of the acute neurological diseases (stroke, seizures/status epilepticus, meningoencephalitis, and spinal cord injury) defined in inclusion criteria between November 2017 and July 2020.

#### Control group (pre-implementation)

Patients included in the control group were admitted and treated with one of the above mentioned acute neurological diseases in the same cooperating regional hospitals between November 2014 and May 2017, i.e. before start of the ANNOTeM implementation.

### Comparisons

#### Comparison of control and intervention period in cooperating regional hospitals

Patients with a diagnosis of a defined acute neurological disease and admitted to the cooperating regional hospitals during the intervention period from November 2017 to July 2020 are compared with patients of the same diagnosis group during the control period from November 2014 to May 2017 before the start of the intervention.

#### Comparison with non-participating hospitals

In order to investigate whether differences in outcome in the cooperating hospitals may be caused by temporal trends, the composite primary outcome will also be analysed for the same two periods in patients fulfilling the identical inclusion criteria but being treated in hospitals of the same region that do not participate in the network (non-network community hospitals). The non-network hospitals were matched according to geographical location, size and amount of acute neurological cases per year. Data for this comparison are also provided by the three insurance companies.

### Study objectives

#### Primary outcomes

The composite primary composite outcome is time to 1) death, **2)** first-time nursing home care or, 3) paid outpatient nursing care for the first time within 90 days after the index event, whichever occurs first.

#### Secondary outcomes

Secondary outcomes are: 1) Treatment proportions for evidence-based therapies such as Stroke Unit treatment, intravenous thrombolysis, endovascular thrombectomy, hemicraniectomy; 2) Hospital transfers; 3) Mortality (Cox regression); 4) Nursing home care; 5) Costs for acute inpatient treatment, hospital transfers, rehabilitation treatment and nursing care.

#### Sample size calculation

Sample size was calculated on the assumption that the implementation of the ANNOTeM concept in the cooperating regional hospitals reduces the rate of dependency from 39% in the control period to 32% in the intervention period, i.e. a relative reduction by one fifth. The assumptions are based on the results of the TEMPiS trial [13].

Patient recruitment started in November 2017 and will run to 31 July 2020. According to the annual case numbers, at least 1820 patients per group with defined inclusion criteria are expected in the eleven cooperating regional hospitals during the intervention period. This sample size ensures a power of 80% (corresponding to a beta of 0.20) and an alpha of 5% for a two-sided test. Case size calculation was performed in nQuery Advisor 7.0.

#### Study time scale

Eligible patients who were admitted to the cooperating hospitals from November 16th 2014 to May 31st 2017 will form the control group. After an implementation period of the ANNOTeM concept, patient recruitment was started in November 2017 in the first intervention hospitals and will continue until July 2020 for primary analysis with 90 days’ follow-up (for details see Table 1). The same periods will be used for the evaluation of time trends in non-network hospitals.

**Table 1:** Start of implementation and recruitment of patients as well as end of recruitment of the intervention hospitals in the study

Intervention hospitals	Start of implementation period	start of recruitment	end of recruitment
BER	10/04/2017	01/02/2018	07/31/2020
LUD	07/02/2017	11/16/2017	07/31/2020
NAU	07/08/2017	11/16/2017	07/31/2020
LUC	08/20/2017	11/18/2017	07/31/2020
KYR	12/11/2017	11/16/2018	07/31/2020
STR	12/23/2017	03/23/2018	07/31/2020
TEM	12/30/2017	03/30/2018	07/31/2020
RAT	10/06/2018	01/04/2019	07/31/2020
UEC	10/16/2018	01/14/2019	07/31/2020
GAR	11/12/2018	02/10/2019	07/31/2020
PRE	10/16/2018	01/14/2019	07/31/2020

**Fig. 2:**
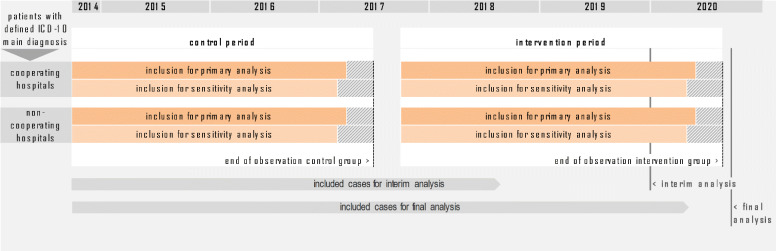
Illustration of the study design of ANNOTeM over time (Study time scale). For the intervention group there is a comparison group both during the intervention period and during the historical reference period

An interim analysis without significance testing is planned after completion of 50% of patient follow-ups in order to inform decision makers about the progress of the scientific evaluation and options for transition into regular care; after the last follow-up is completed end of October 2020, the final analysis is expected in January 2021 (Fig. 2).

### Statistical analyses

#### Primary analysis

Because health insurance data will be provided anonymously, we cannot determine the effect of telemedicine consultation on an individual patient level. Rather, we will evaluate the effects of the ANNOTeM concept in clusters. The primary outcome will be analysed as the time from the index visit to the occurrence of the compound outcome as described above. Separate Cox models will be fitted for eligible patients insured at the cooperating health insurance companies, admitted and treated in both, the pre-implementation period and the intervention period. Cox models will comprise sex (m/f only), age and co-morbidity (Y/N) as covariates. Hazard rate ratios will be used to compare time-to-compound event between the two periods. All tests will be performed at a significance level of alpha = 0.05, adjusted for two-sided testing and the k = 6 tests of the primary and secondary analysis. All tests will be performed using the statistics package ‘R’ (version 3.6).

#### Secondary analysis

The secondary analysis compares the survival rates obtained in the primary for the network clinics with the group of non-cooperating hospitals using the log-rank-test for 2 × 2 factorial designs (control vs intervention period and network vs non-network hospitals). A treatment effect will be revealed by a significant interaction in favour of the network clinics during the intervention period.

#### Sensitivity analysis

In a sensitivity analysis, we extend the follow-up of the composite primary outcome to 120 days after the index event. Secondly, we perform separate analyses for the outcomes death, first-time nursing home care and paid nursing care for the first time, which together make up the composite endpoint in the primary analysis. Thirdly, we perform a subgroup analysis of the composite primary outcome for following groups: patients with stroke vs other defined acute neurological diseases; patients with vs without nursing care before the index event; patients in cooperating hospitals with vs without neurological departments or telemedicine support before the start of the ANNOTeM intervention. Finally, we investigate patients in the cooperating hospitals with traumatic brain injuries who cannot be included in the main intervention group as a companion study population.

### Data collection, management, and analysis

#### Data collection methods

The health insurance data for determining the defined outcomes are provided by the cooperating statutory health insurance companies (AOK Nordost, BARMER, TK) in accordance with the defined inclusion criteria. Within the framework of ANNOTeM, pseudonymised personal data of the health insurances will be transmitted in accordance with §75 Social Security Code V to a third trust party, which forwards the data in anonymised form to the evaluating institute. This forwarding has been approved by the supervisory authorities of the participating health insurance companies.

In addition to the account data for identifying the defined outcomes and costs, basic data on age and sex, insurance status and paid nursing care or nursing home care are provided at the beginning of observation.

#### Data management

The manual for data management was approved by TMF (Technologies, Methods and Infrastructure for Networked Medical Research) and the data protection officer of the Charité.

## Discussion

Ageing populations along with structural deficits in underserved regions of North-East Germany [17] with an increasing shortage of medical specialists are major challenges in providing state-of-the-art treatment for acute neurological patients. At the same time, there is a need for comprehensive access to new, evidence-based therapies and standardized operating procedures for neurological emergencies in community hospitals, mostly without a neurology department. New initiatives to combine medical expertise with the use of modern infrastructural components have been established within the framework of telemedicine networks. So far, evidence for improving the quality of care is only available from acute telestroke projects. Hence, there is a need for large-scale studies that examine the benefits of such networks for outcomes and cost-effectiveness. ANNOTeM evaluates clinical outcomes for patients with acute neurological diseases including but not exclusively stroke treated with telemedicine support. ANNOTeM aims to establish and sustainably improve regional structures by continuous knowledge transfer to all professional groups involved in the care of acute neurological patients in the regional hospitals by continuous training and the introduction of standardized processes.

We acknowledge several limitations of the study: Changes in the care of patients with effects on the primary outcomes measured here may occur independently of study design through health policy measures and health care innovations. This can result in an overestimate of improved care for the intervention group compared to the pre-implementation control group. In order to control for this possible time trend, we have introduced a parallel control group of patients treated in non-network hospitals. A further limitation is that the definition of part of the composite outcome is based on routine data of the health insurances. It is possible that patients or their relatives do not apply for home care support provided by the health system even when they have a respective need of assistance. In addition, the decision for home care also depends, among other things, on the available social support at home or personal preferences. However, previous analyses have shown that the parameters of care support and home care correlate well with the degree of disability [18]. The approach taken in this project will be evaluated in the setting of the German health care system and results can therefore not be generalized.

On the other side, the study has several important strengths. First, the total number of patients treated in the hospitals with regard to the main diagnoses is recorded on the basis of health insurance data. Thus, the bias of patient selection by informed consent procedures often present in clinical studies is minimized. Second, study outcomes are assessed independently. Third, the combination of data collected within the telemedicine consultations along with quality management of the health insurance data provide the opportunity to evaluate the effects with a direct link to the health care context.

### Study status

The intervention started in November 2017 and results are expected in January 2021.

## Data Availability

Not applicable.

## Abbreviations

AWMF: Joint Workgroup of German Medical Societies (Arbeitsgemeinschaft der Wissenschaftlichen Medizinischen Fachgesellschaften)
CT: Computer tomography
DGN: German Neurological Society (Deutsche Gesellschaft für Neurologie)
GDPR: General Data Protection Regulation
ICD: International classification of diseases
ITT: Intention-to-Treat
MRI: Magnetic resonance tomography
SOP: Standardized operating procedures
TMF: Technologies, Methods and Infrastructure for Networked Medical Research
